# Real-time measurement of *Plasmodium falciparum*-infected erythrocyte cytoadhesion with a quartz crystal microbalance

**DOI:** 10.1186/s12936-016-1374-7

**Published:** 2016-06-13

**Authors:** Daniela Kömpf, Jana Held, Stefani F. Müller, Hartmut R. Drechsel, Serena C. Tschan, Hinnak Northoff, Benjamin Mordmüller, Frank K. Gehring

**Affiliations:** Biosensor Research Group, Institute of Clinical and Experimental Transfusion Medicine, University Hospital of Tübingen, Tübingen, Germany; Institute of Tropical Medicine, University of Tübingen, Tübingen, Germany; DZIF–Deutsches Zentrum für Infektionsforschung, Standort Tübingen, Germany; State Health Office Baden-Württemberg, Stuttgart, Germany; 3T GmbH & Co KG, Tuttlingen, Germany

**Keywords:** Malaria, PfEMP1, Cytoadhesion, Thickness shear mode sensors, Biosensor, CSA, CD36

## Abstract

**Background:**

An important virulence mechanism of the malaria parasite *Plasmodium falciparum* is cytoadhesion, the binding of infected erythrocytes to endothelial cells in the second half of asexual blood stage development. Conventional methods to investigate adhesion of infected erythrocytes are mostly performed under static conditions, many are based on manual or semi-automated read-outs and are, therefore, difficult to standardize. Quartz crystal microbalances (QCM) are sensitive to nanogram-scale changes in mass and biomechanical properties and are increasingly used in biomedical research. Here, the ability of QCM is explored to measure binding of *P. falciparum*-infected erythrocytes to two receptors: CD36 and chondroitin sulfate A (CSA) under flow conditions.

**Methods:**

Binding of late stage *P. falciparum* parasites is measured in comparison to uninfected erythrocytes to CD36- and CSA-coated quartzes by QCM observing frequency shifts. CD36-expressing cell membrane fragments and CSA polysaccharide were coated via poly-l-lysine to the quartz. The method was validated by microscopic counting of attached parasites and of erythrocytes to the coated quartzes.

**Results:**

Frequency shifts indicating binding of infected erythrocytes could be observed for both receptors CD36 and CSA. The frequency shifts seen for infected and uninfected erythrocytes were strongly correlated to the microscopically counted numbers of attached cells.

**Conclusions:**

In this proof-of-concept experiment it is shown that QCM is a promising tool to measure binding kinetics and specificity of ligand-receptor interactions using viable, parasite-infected erythrocytes. The method can improve the understanding of the virulence of *P. falciparum* and might be used to cross-validate other methods.

**Electronic supplementary material:**

The online version of this article (doi:10.1186/s12936-016-1374-7) contains supplementary material, which is available to authorized users.

## Background

Quartz crystal microbalances (QCM) are highly sensitive sensors that can reliably weigh material in the nanogram range and are highly sensitive to changes in biomechanical properties of coupled biomaterial. They consist of a thin, usually round slice of crystalline quartz with a gold electrode on each side. Oscillating crystals are composed of α-Quartz (SiO_2_) that is known to be piezoelectric material, characterized by: (a) appearance of electrical potential when it is subjected to mechanical stress (piezoelectric effect); and, (b) deformation of the material when it is subjected to electrical potential (inverse piezoelectric effect). Due to the piezoelectric character the quartz is stimulated to oscillations when connected to AC voltage [1]. Material attaching to the quartz’s surface reduces the frequency. Thus, an increase in weight leads to an adequate decrease in frequency. In biosciences, mass-sensitive QCM are already well accepted, while applications for clinical questions, in particular with whole blood samples, are novel and have not yet been exploited. Lately, this technology has been applied to the typing of blood groups, detection of bacteria, bacterial toxins, viruses, and measuring affinity of antibodies. Further studies on adherent cell types have been performed but the measurement of whole cell interactions of primarily non-adherent cells has not been addressed thoroughly [1–6].

Here a novel QCM-based assay is presented to evaluate whole cell interactions by measuring cytoadhesion of erythrocytes infected with the malaria parasite *Plasmodium falciparum* to endothelial cell receptors. Malaria is the most important parasitic infection and accounted for more than 200 million episodes and 438,000 deaths in 2015 [7]. The disease is caused as a result of the infection by protozoan parasites of the genus *Plasmodium.* Symptoms only occur during the blood stage of the infection when parasites multiply in red blood cells. Among five different species known to infect humans, *P. falciparum* causes the most severe form of the disease and is responsible for the vast majority of deaths. In contrast to the other species, *P. falciparum*-infected red blood cells (iRBCs) adhere to vascular endothelium of postcapillary venules (sequestration) and to non-infected erythrocytes (rosette formation) in the second half of their asexual replication, cycle via *P. falciparum* erythrocyte membrane protein 1 (PfEMP1), which is expressed on the surface of iRBCs [8, 9]. Through cytoadhesion the parasite avoids clearance by the spleen; this is an immune evasion mechanism that can lead to excessive parasite multiplication and may result in complications such as cerebral and placental malaria [10–13]. Important receptors involved in this process are CD36, ICAM1 in cerebral malaria and chondroitin sulfate A (CSA) in placental malaria [14–16]. This work focuses on the binding of iRBC to CD36 and CSA. CD36 is expected to be the main receptor of iRBC adhesion as nearly all laboratory isolates of *P. falciparum*, as well as clinical isolates from patients attach to this receptor [15–17]. CSA on the other hand, is well described to be the main receptor of parasite binding in placental malaria and parasites expressing the highly conserved PfEMP1 gene *var2csa* are known to bind to this polysaccharide [14, 18, 19]. Placental malaria can cause low birth weight of newborns, premature delivery, abortion, stillbirth, maternal anaemia, as well as infant and maternal morbidity and mortality [20, 21]. CSA is also present on other endothelial tissue where parasites might bind to it [22].

Different methods have been developed to measure PfEMP1-mediated cytoadhesion. Up until now most of the established cytoadhesion assays have been performed under static conditions [23–26], thus lacking shear stress which is assumed to be important for the parasites to get in contact with the appropriate receptors. However, flow-based assays exist as well and some include static periods at the beginning of the measurement to allow settling of parasites, so that they are able to make contact with the immobilized receptors [27–32]. The disadvantage of some of these systems is that handling of the constructions is cumbersome; for example, the read-out is microscopy-based and therefore analysis of results is less standardized [33–36]. Hence, a real-time detection of cytoadhesion is not possible and an adequate evaluation of the experiments performed with these microscope-based assays is time consuming and often reader biased. Another limiting aspect is that measuring of binding times and kinetics as well as discrimination between loosely adsorbed and truly adhered cells are very difficult.

The analysis of this complex interaction would benefit from other highly sensitive and reproducible methods based on a different technology for validation. Therefore, QCM is adapted in this proof-of-concept experiment for the analysis of PfEMP1-mediated iRBC binding to the cell surface of CD36-expressing melanoma cells as well as to CSA. It is the first time that QCM under flow conditions has been used with viable iRBCs and these experiments show the great potential of this approach for future studies in cell–cell as well as cell-receptor studies.

## Methods

### Parasite culture

The parasite strains FCR3-CD36 and FCR3-CSA were kindly provided by Artur Scherf, Institute Pasteur Paris, and were cultivated as previously described [37] in fresh group O+ erythrocytes at 5 % haematocrit in RPMI-1640 supplemented with 2 mM l-glutamine, 50 µg/ml gentamycin, 25 mM HEPES, 0.5 % Albumax II, and 5 % AB+ human serum (complete medium). Culture flasks were aerated with 5 % carbon dioxide, 5 % oxygen and 90 % nitrogen. Specific binding phenotype of parasites was maintained by regular selection. For CD36 this was performed by monthly panning of late-stage, infected erythrocytes on C32-melanoma cells, at pH 6.8, as described elsewhere [38, 39]. To select parasites for specific binding to CSA, late blood stages were panned monthly on polystyrene six-well plates, coated with CSA from bovine trachea (Sigma) as previously described [40]. No negative selection of parasites was done and parasites were used for experiments after at least one round of selection. Prior to adhesion assays, cultures were synchronized by treatment with 5 % sorbitol and late stages were purified/enriched by magnetic cell sorting (MACS) as described elsewhere [41].

### C32-melanoma cell culture

C32-melanoma cells expressing large amounts of CD36 were obtained from the American Type Culture Collection (ATCC) and cultured at 5 % carbon dioxide and 37 °C using DMEM supplemented with 50 µg/ml gentamycin, 10 % FCS, 2 mM l-glutamine, and 1 % MEM non-essential amino acid solution (NEAA). Splitting of cells was performed when cultures reached 80–90 % confluence.

### QCM sensors

The QCM sensors used in this work were AT-cut, 10 MHz quartz crystals (8 mm in diameter, thickness of 166 μm (KVG Quartz Crystal Technology, Neckarbischofsheim, Germany) with a special gold electrode design developed by FKG.

### Base coating of quartz surface

To successfully immobilize receptors to the quartz, a base coating with poly-l-lysine (PLL, Fluka 70–150 kDa) to untreated sensors was done by dropping 60 µl of 0.5 mg/ml PLL in H_2_O to the quartz and subsequent drying under a stream of nitrogen [25]. For each experiment a new quartz was used as a complete cleaning of used quartzes was not possible.

### Coating of CD36 to the quartz surface

CD36 belongs to the class B scavenger receptor proteins and is characterized by a special structure that is necessary to ideally present the specific binding site to parasitized erythrocytes. Thus, to preserve this structure for CD36 coating, membranes or membrane fragments of C32 cells were prepared by homogenization of C32 cells with a potter on ice using a fractioning buffer consisting of Tris/HCl (50 mM), NaCl (150 mM) and EDTA (1 mM). To separate the membrane fragments from nuclei and intact cells, the suspension was centrifuged at 540×*g* (10 min, 4 °C). In a next step the supernatant was centrifuged at 2500×*g* (15 min, 4 °C) to remove further organelles. The last centrifugation step using an ultracentrifuge at 100,000×*g* (1 h, 4 °C) served to concentrate the membrane fragments. After removal of the supernatant the pellet was re-suspended in 400 µl fractioning buffer supplemented with 800 µl of a proteinase-inhibitor solution (Roche). Aliquots were stored at −20 °C. To achieve a coating as homogeneous as possible, membrane suspensions were incubated with PBS-Tween-20 (0.1 %) for 5 min. Immobilization on PLL-coated quartzes was done by dropping 60 µl of suspension to the quartz, and subsequent incubation for 10 min before unbound membranes were washed away with PBS. This was followed by fixation with 4 % paraformaldehyde in PBS on ice for 7 min and blocking with 2 % BSA in PBS (1 h, RT).

### Coating of CSA to the quartz surface

To coat CSA to the quartz surface CSA (Sigma, 1 mg/ml in PBS) was immobilized ‘online’ to PLL. Following the insertion of the PLL-coated quartz to the QCM platform, the CSA solution was allowed to run over the quartz at a flow rate of 50 µl/min for 10 min to enable CSA to bind to the PLL-coated quartz. This was followed by a washing step with flow medium for 5 min at 100 µl/min.

To verify binding specificity for PLL basis coating, control human serum was added to the quartz outside of the platform before adding iRBCs (FCR3-CSA) without prior coating of CSA.

### Verification of coating by immunofluorescence

Distribution of the receptors CD36 and CSA on the quartz were verified by an indirect immunofluorescence test (IFT). A separate sample was treated with secondary antibody only, to control for unspecific binding of the antibody. After coating, quartzes were fixed with 4 % paraformaldehyde in PBS on ice for 7 min, followed by blocking with 2 % BSA in PBS (1 h, RT).

The distribution of CD36 on the membrane of C32 cells was visualized by staining with a monoclonal mouse-anti CD36 antibody (Abcam, 1:10 in PBS, 1 h, RT); and a sheep anti-mouse IgG F(ab′)2 fragment–FITC antibody (Sigma, 1:30 in PBS, 1 h, RT). For verification of CSA-coating, quartzes were stained with a monoclonal mouse anti-CSA antibody (Sigma, 1:100 in PBS, 1 h, RT) and a FITC-labelled goat anti-mouse IgM antibody (Sigma, 1:128, 1 h, RT).

Pictures of stained quartzes were taken by the digital camera Casio QV 5700 at the Axioskop 2MAT microscope with 100× or 500× magnification. Overlay of pictures was made with Adobe Photoshop 7.0.

### Sensor platform and adhesion assay using QCM-sensors

**Fig. 1:**
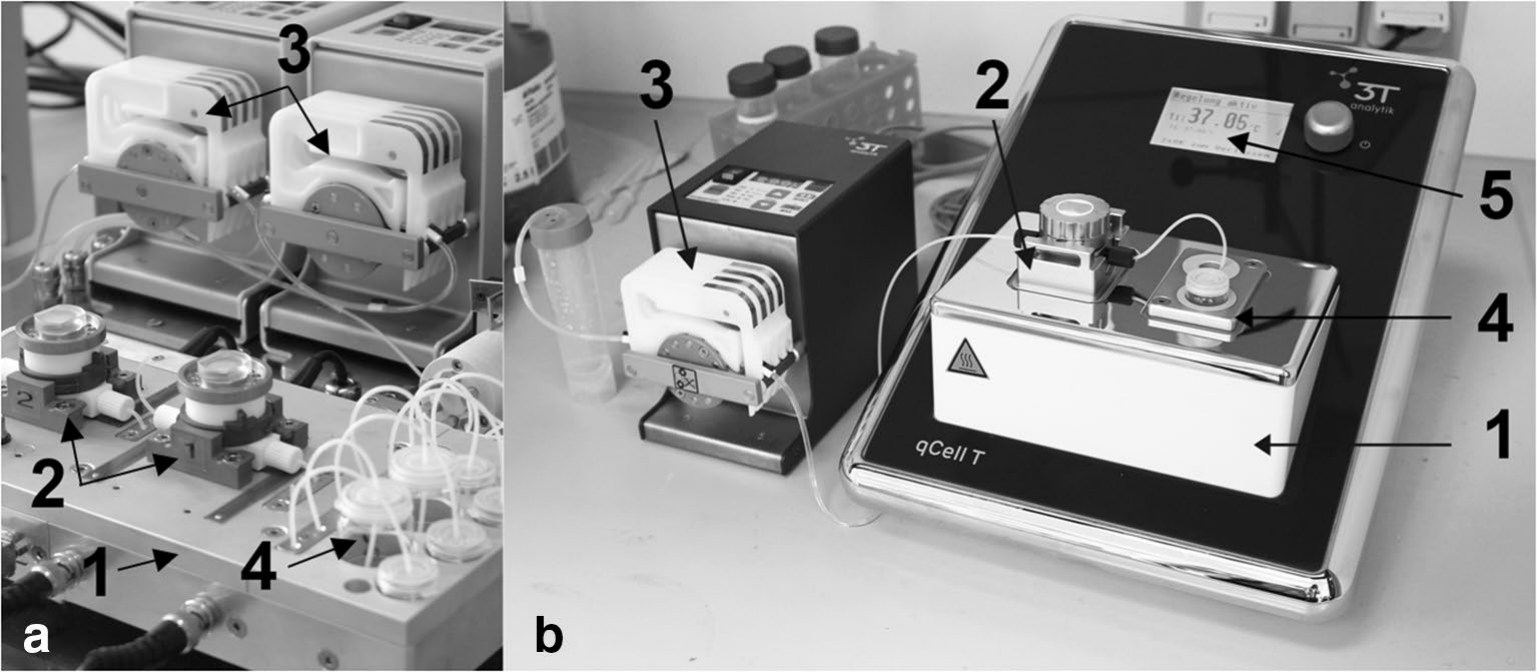
QCM platform settings. **a** *F*id*g*et Type 1 and **b** qCell T. Thermo-controlled QCM platform (*1*) with fluidic measuring chambers for QCM-sensors (*2*); peristaltic pumps (*3*); fluidic system with sample retainers (*4*); and, digital display (*5*)

QCM-sensors can detect very low mass changes deposited on the quartz by changes in resonance frequency. Frequency shifts due to adhesion processes to receptors were electronically recorded and analysed using a sensor platform called ‘*F*id*g*et Type 1’ [42, 43] (Fig. 1a). As two integrated QCM sensor channels exist, it is possible to simultaneously examine two samples (sample + control) under the same conditions. The platform is equipped with an automated flow-injection system and a Peltier element, which enables stable measurements at specific temperatures. Control measurements with human serum focusing on the PLL coating systems were performed at a later time point on the further developed and commercially available beta version sensor platform qCell T [44] (Fig. 1b). The one channel platform qCell T provides dissipation measurements in addition to the frequency curves. However, as dissipation curves do not add substantial information to the results they are not presented here. The other functions and results obtained from the measurements are comparable to the old version but stability, sensor handling and software have been essentially improved.

To ensure a sterile environment for measurements, the fluidic system was first disinfected by incubation with 1 % sodium hypochlorite for 10 min. After washing with sterile distilled water and a de-gassed flow medium (complete medium without sodium hydrogen carbonate and without human serum), sensors were inserted into the pre-heated platform (37 °C). Cell suspensions were diluted in flow medium and were transferred to the sensor surface at a continuous flow rate (50 µl/min).

The optimal parameters for detection of adhesion processes to the receptors by iRBCs were achieved by systematically adapting measurement periods, cell concentrations and flow rates. Final experiments with CD36 and CSA coated to PLL were performed with the optimized closed fluidics system. The flow rate was adjusted to 50 µl/min to fill the fluidic system at the onset of the measurement with flow medium, and to pump the cell suspension over the sensor during the period of the whole measurement of cell adhesion. For the assays, 1.5 × 10^5^ iRBCs/ml or RBCs/ml were diluted in flow medium and allowed to circulate and flow over the quartz at 50 µl/min for 2.5 h in a closed system. The inner diameter of the tubes used was 0.38 mm, resulting in a velocity of 73 mm/s for the used flow rate.

### Verification of parasite adhesion by microscopy after QCM

After binding experiments, quartzes were removed, rinsed with flow medium and fixed with 4 % paraformaldehyde in PBS on ice for 7 min. To determine the stage and viability of iRBCs adherent to the sensor’s surface, samples were stained with the DNA-stain DAPI (Sigma, 0.2 µg/ml, 5 min, RT). The mean number of bound iRBCs and RBCs of three visual fields, corresponding to a surface of 22.1 × 10^3^ µm^2^, were determined by counting under a microscope (AxioImager, Zeiss, magnification 500×).

### Analysis

Frequency shifts were analysed using qCell T software. Median differences, the interquartile range as well as the single measurements are shown for frequency shifts and the subsequent microscopic count data of attached parasites to the quartz. Microscopic counts of attached infected and uninfected RBCs are correlated to frequency shifts by Spearmans rho for paired samples.

## Results

### Verification of CD36 coating of the quartz

**Fig. 2:**
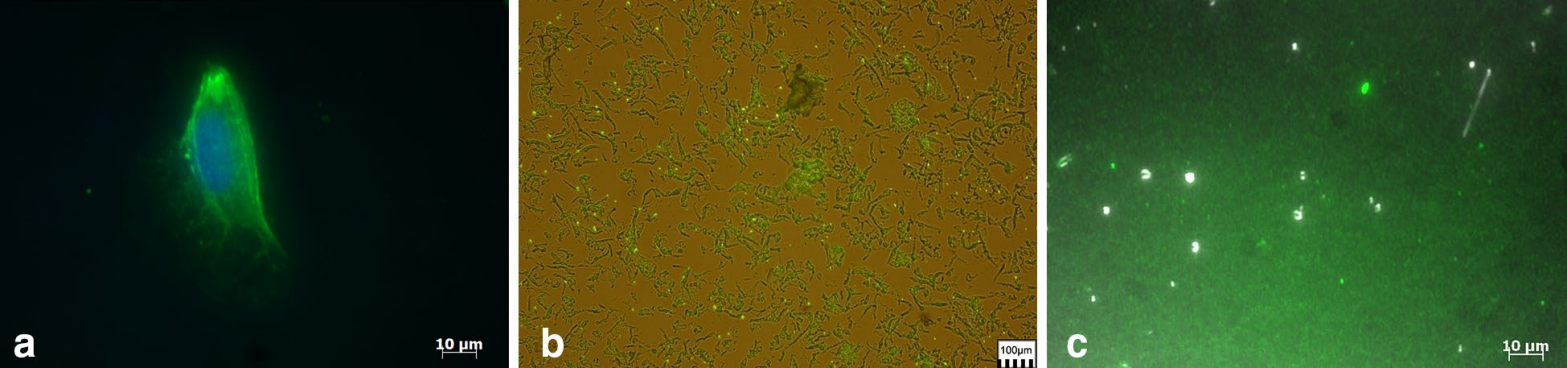
Quartz coating with CD36 using membranes of melanoma cells and CSA via PLL. **a** Overlay picture of a C32 melanoma cell stained for DNA (DAPI, *blue*) and CD36 (FITC, *green*) showing a homogenous spread of CD36 on the surface and the nucleus in the centre of the cell; **b** fluorescence picture of isolated and homogenized cell membranes of C32 melanoma cells immobilized to a PLL-coated quartz and stained with a CD36 antibody (FITC, *green*); **c** overlay picture of a quartz coated with PLL/CSA showing an even distribution of fluorescence signal, indicating a homogenous coating of CSA to the quartz. The quartz was stained with an anti-CSA antibody (FITC, *green*)

Expression of CD36 on C32 melanoma cells was verified by fluorescence microscopy, after staining with an anti-CD36 antibody showing an intense staining evenly spread over the surface of the cell (Fig. 2a). Isolated cell membranes of melanoma cells were immobilized to the quartz surface via PLL and were stained with an anti-CD36 antibody.

Images obtained by fluorescence microscopy show that an almost homogenous coating of the quartzes with CD36 could be achieved and that the CD36 antibody epitope was not destroyed by the processes of cell homogenization and the following differential centrifugation (Fig. 2b). Minor differences in the dissemination of membranes to the sensors are caused by the nature of the biological medium in which CD36 is embedded. To preserve the complex structure of the CD36 protein, which belongs to class B scavenger receptor proteins, whole membranes of C32 melanoma cells are used. Whole cells could not be used as the layer on the sensor would be too thick and not rigid enough to determine interactions of the parasites with the coated sensors.

### Verification of CSA coating via fluorescence microscopy

Coating of the quartz sensor with the receptor was confirmed by fluorescence microscopy (Fig. 2c). CSA immobilized via PLL resulted in a homogenous and dense coating, illustrated by a strong, specific fluorescence signal. Coating of the sensor with CSA via PLL was additionally verified by on-line measurement in the platform. Injection of CSA led to a strong binding signal, which remained stable even after rinsing with high flow rates. Further incubation steps with different concentrations of bovine serum albumin (BSA) showed no additional binding events (Additional file 1). Thus, potential unspecific binding sites appear to be non-accessible.

### Measurement of binding of *Plasmodium falciparum* iRBCs to receptors (CD36 and CSA) via QCM

**Fig. 3:**
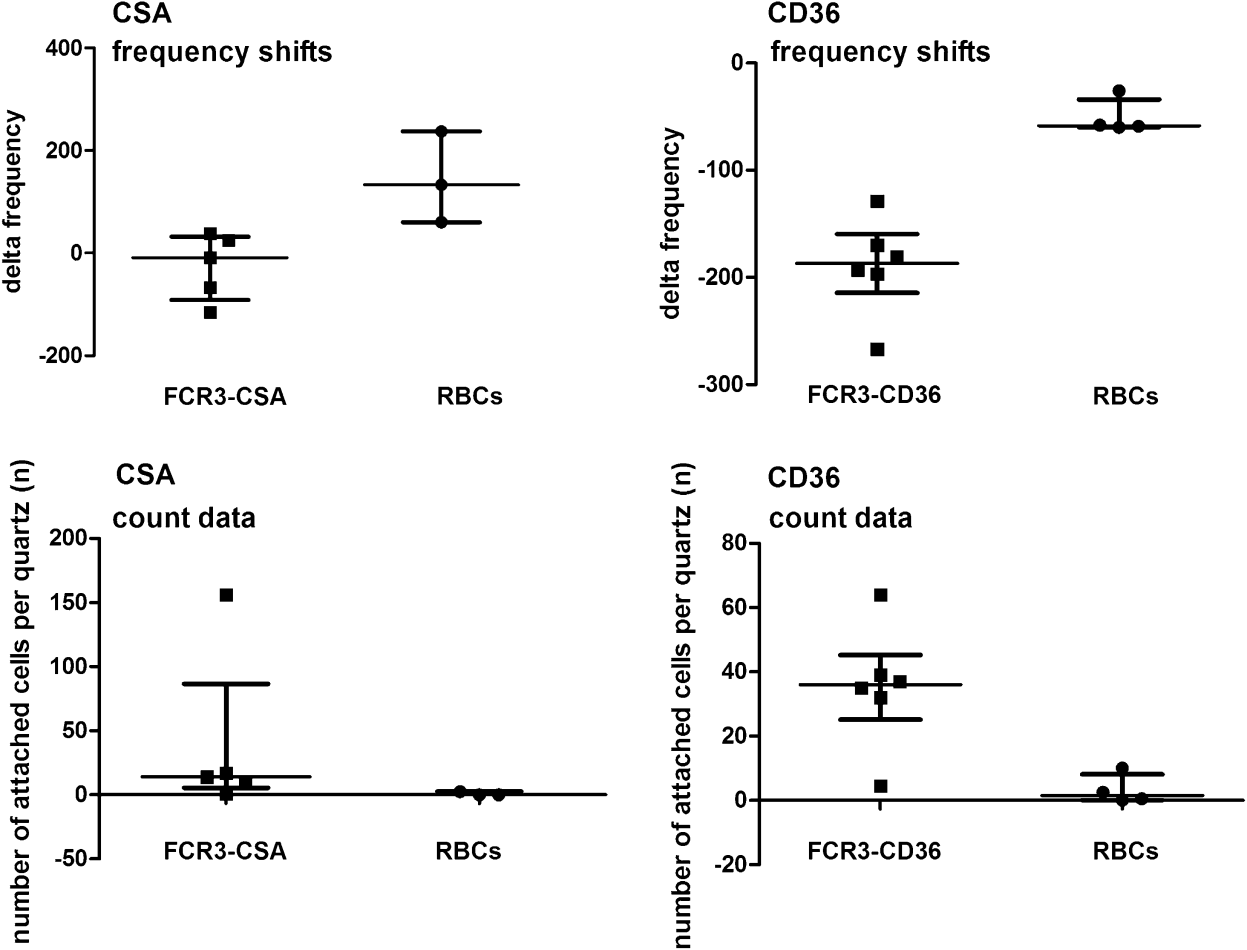
Overview of QCM experiments measuring attachment of cells to receptors by frequency shifts and subsequent microscopic count. Median, interquartile range and single measurements of frequency shifts measured by the QCM platform *F*id*g*et Type 1 (*upper panel*) and subsequent microscopic count of the number of attached cells per microscopic field (*lower panel*) for either *P. falciparum* iRBCs of FCR3-CSA strain (n = 5) and red blood cells (RBC, n = 3) added to CSA receptors (CSA), or for iRBCs of FCR3-CD36 strain (n = 6) and RBCs (n = 4) added to CD36 receptors. Results show that iRBCs (FCR3 parasites) bind to the respective receptor, reflected by a dampening of the frequency and a higher count data in the microscope whereas RBCs do not bind. *CSA* chondroitin sulfate A, *PLL* poly-l-lysin

In Fig. 3 an overview of the frequency shifts reflecting the mass change due to binding is shown in comparison to the microscopic counts of attached cells on the quartz. Parasites selected for CD36 binding (FCR3-CD36) adhered to CD36, reflected by a dampening of the signal (median frequency shift: −187 Hz, n = 6), whereas uninfected RBCs did not adhere to CD36 (median frequency shift: −58.5 Hz, n = 4). This was also reflected by the microscopic cell count showing that a median of 36 iRBCs (n = 6) attached to one visual field of the CD36-coated quartz, whereas only a median of 1.5 RBCs (n = 4) attached in the control experiment. The parasite strain selected for CSA binding (FCR3-CSA), specifically adhered to CSA immobilized to PLL, reflected by a dampening of the signal (median frequency shift: −9 Hz, n = 5) after adding of parasites to the flow system. Uninfected RBCs did not bind (median frequency shift: 133 Hz, n = 3). The results are reflected by the parasite counts performed after the experiments. When checking for attached parasites a median of 14 iRBCs (n = 5) could be counted in one visual field per quartz compared to a median of 0 attached RBCs (n = 3) on the control quartz. For the measurement of RBCs on the CSA-coated quartz, an increase in frequency occurred which is caused by a so-called ‘missing mass effect’ [45] leading to alterations in visco-elasticity.

**Fig. 4:**
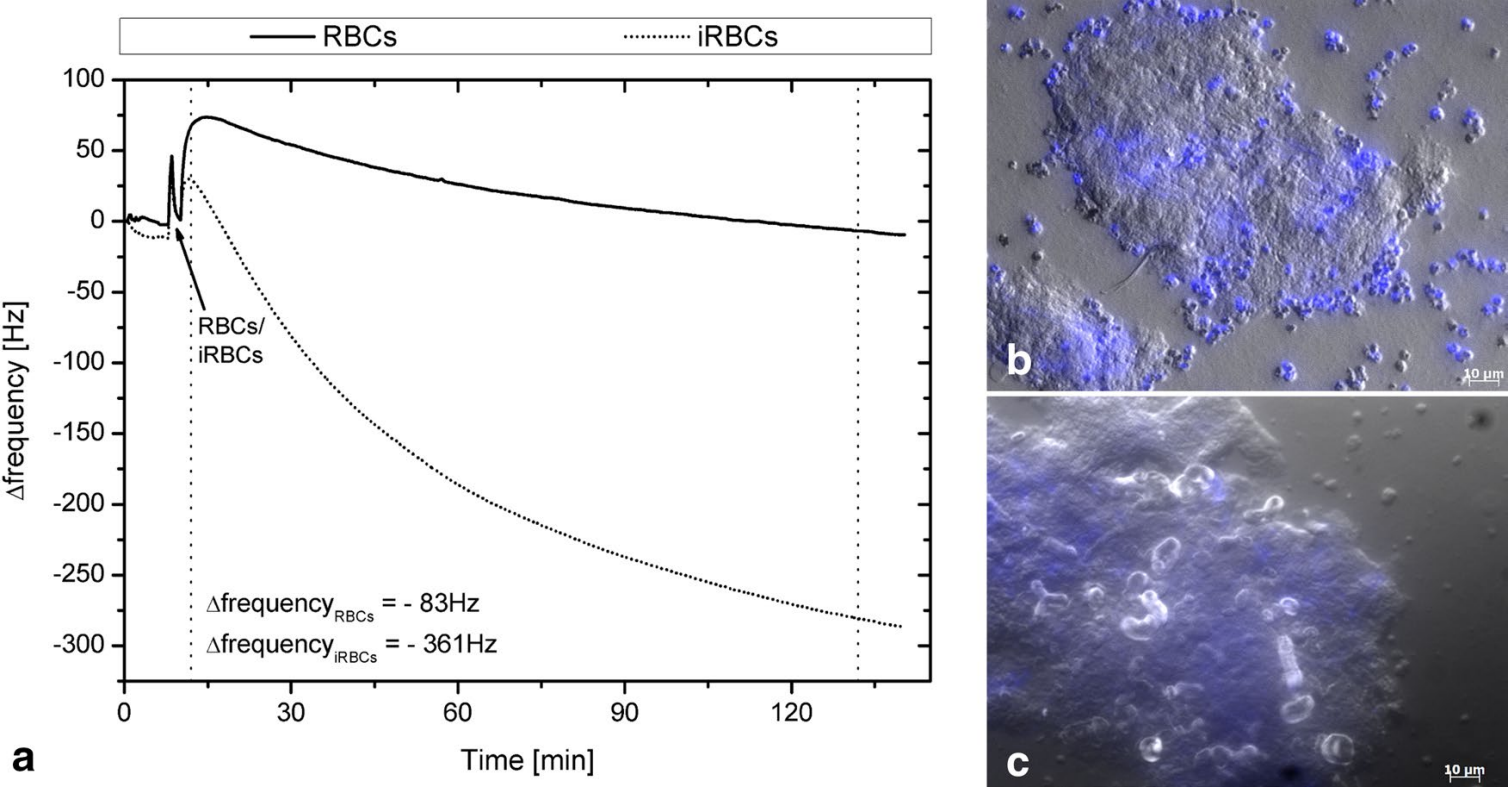
Example QCM signals and pictures obtained for CD36. Measurements were carried out on the two-channel sensor platform *F*id*g*et Type 1. **a** The samples (iRBCs and RBCs) were injected after achievement of a stable baseline followed by a short stop-flow interval. Adhesion of iRBCs (FCR3-CD36) led to a decrease of the signal. The low frequency shift obtained with RBCs shows an unspecific drift due to the alterations in the viscosity of the fluid. Frequency shifts after 2.5 h (time frame indicated by *vertical dotted lines*) are −83 Hz for RBCs and −361 Hz for iRBCs indicating the attachment of iRBCs to CD36 on the quartz; **b** and **c** pictures of corresponding quartzes stained with DAPI after measurement in the QCM platform confirming the results. After injection of iRBCs it can be clearly seen in (**b**) that iRBCs bind to the attached C32 cell membranes expressing CD36 on the quartz. In contrast in (**c**), after the experiment with RBCs only very few RBCs are detected on the sensor surface and only cell membranes can be seen

**Fig. 5:**
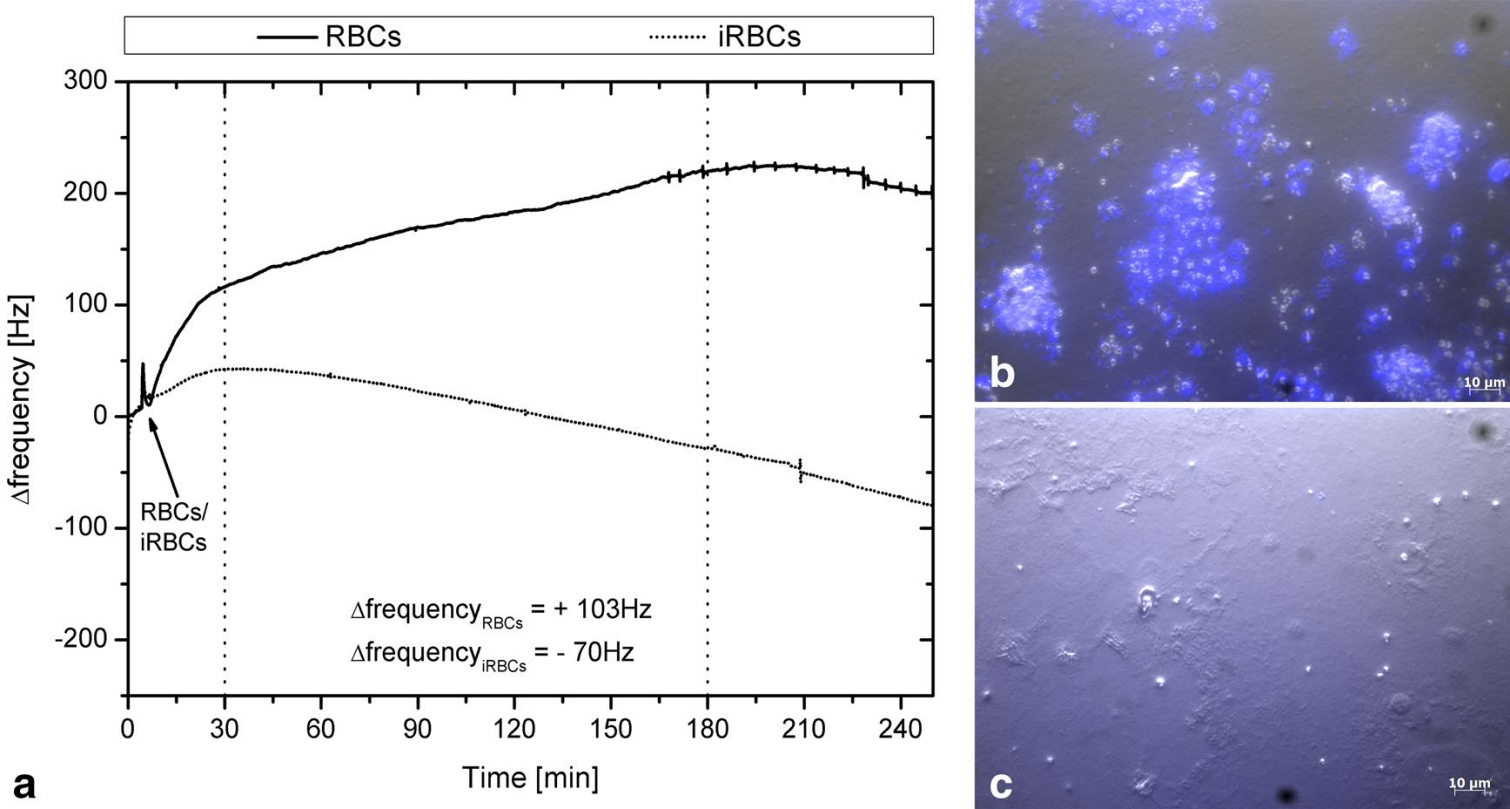
Example QCM signals and pictures obtained for CSA via PLL. Measurements were carried out on the two-channel sensor platform *F*id*g*et Type 1. **a** Example *curves* show the frequency changes (∆f) during the course of the experiment. After injection of the samples (iRBCs and RBCs), adhesion of iRBCs (FCR3-CSA) led to a decrease of the frequency signal, whereas the frequency signal for RBCs showed no signs of attachment. Frequency shifts after 2.5 h (time frame indicated by *vertical dotted lines*) are +47 Hz for RBCs and −63 Hz for iRBCs indicating attachment of iRBCs to the CSA; **b** and **c** pictures of the corresponding quartzes stained with DAPI after measurement in the QCM platform confirm the results. In **b** one can see the quartz after the experiment with iRBCs showing the stained nuclei of the attached iRBCs to the CSA on the quartz. In **c** the quartz after the experiment with RBCs can be seen, showing only very few RBCs on the sensor surface

Addition of late stage iRBCs to CD36 and late stage iRBCs to CSA led to a strong frequency shift compared to RBCs only. Representative frequency shifts obtained with infected and uninfected RBCs for a PLL-CD36-coated quartz are shown in Fig. 4a and for a PLL-CSA-coated quartz in Fig. 5a. Addition of infected or uninfected RBCs led to a short peak due to stopping of the pumps at the beginning of the measurement. Starting and stopping of the pumps led to differences in frequencies that restored when the normal flow rate (50 µl/min) was applied. After the short peak observed at the beginning of the measurement, a decrease in frequency appeared caused by the cells arriving on the sensor’s surface. Thus, the sensors detected the occurring mass change. The continuous flow of medium over the sensor rapidly spread the cells over the sensor and unbound cells were washed away leading to a balance of the frequency (≤5 min). The slow decrease of frequency over the 2.5 h was due to the continuous adhesion process of parasites during the experiment. When adding RBCs, the signal stayed at the same level or even increased. The increase for RBCs seen in Fig. 5a was due to the visco-elastic behaviour of the PLL/CSA layer, caused by the missing mass effect, mentioned above. Differences between the course of the two signals of CD36 and CSA are due to the differently coated quartzes; for CD36, whole cell membrane fragments were immobilized to the quartz surface whereas for CSA the quartz was coated with the polysaccharide alone. As it is assumed that iRBCs develop stronger adhesion to CD36 than to CSA, different binding kinetics can be observed.

The number of attached cells was correlated with the observed frequency shift (rho = −0.55, p = 0.017). Example images of one microscopic field are given in Fig. 4b, c for CD36 and iRBCs and RBCs, respectively, and in Fig. 5b, c for CSA and iRBCs and RBCs, respectively. They show that iRBCs with FCR3-CD36 parasites attached to CD36-coated quartzes and that iRBCS with FCR3-CSA parasites attached to CSA-coated quartzes (Figs. 4b, 5b), in contrast to uninfected RBC (Figs. 4c, 5c), where only a very few cells attached.

PLL by itself is very sticky for any kind of cell and can therefore not be used without a receptor/analyte. PLL-coated quartzes alone bound added cells very strongly and quickly (within minutes) (see Additional file 2 for RBCs). As a separate control experiment, human serum proteins were immobilized to PLL and as expected, frequency shifts showed that iRBCs (FCR3-CSA) did not bind to human serum proteins [median frequency shift: 46 Hz (n = 3), Additional file 3].

## Discussion

Measuring with QCM allows real-time observation of cellular interactions by evaluating adhesion processes of intact *P. falciparum*-infected RBCs and their respective receptor. Here a novel method is presented to measure cell adhesion processes of iRBCs to two different receptors under flow conditions. Binding is analysed of iRBCs to CD36, a receptor most *P. falciparum* isolates adhere to, and binding to CSA, the main receptor involved in placental malaria. Dampening of the signal showed that iRBCs bound with higher affinity to the receptor when compared to uninfected RBCs, corresponding with the results obtained by microscopic counting of attached cells. Control experiments with human serum as unspecific analyte showed that iRBCs did not bind to serum proteins coated to PLL. The observed increase in frequency similar to the experiments of CD36 and RBCs is probably caused by a change of the visco-elasticity of the sensors surface called the missing mass effect [45]. Only by adding cells and medium to the sensor, could a conformation change of PLL be achieved so that a more rigid layer is present, influencing the sensor’s behaviour. However, a control experiment with PLL and human serum shows that frequency shifts obtained with the iRBCs using the PLL/CD36- or PLL/CSA-coated sensors are due to specific interactions between the iRBCs and the receptors, and not to unspecific interactions with PLL. Parasite binding to CSA is a relevant model for placental malaria, and evaluation of inhibiting antibodies and vaccine candidates under flow conditions by QCM could complement existing methods. Further experiments with the QCM should be performed to explore the potential of this methodology in studying malaria parasites in more depth. Future experiments should include different parasite strains, including freshly isolated clinical isolates from the relevant organ (e.g., placenta), in addition experiments with inhibiting antibodies and possible blocking proteins should be performed to evaluate the potential of the methodology. One weakness of the system is that coating of a sugar as done with CSA might not represent the three-dimensional structure found in nature, a problem circumvented by coating of membranes displaying CD36 on their surface. Despite this however, this methodology has the weakness that one cannot fully control correct positioning of the membranes, and some might even be attached upside down. So far QCM technology has been applied only to analyse molecules in malaria research but not for studying the whole parasite. One group established the differentiation of *Plasmodium* species by analysing DNA fragments [46]. Another group evaluated the kinetics of protein interactions on the example of binding kinetics of the PfEMP1 protein var2CSA to different antibodies [47]. Here for the first time whole cell interactions of live parasites are investigated. This opens possibilities for malaria research not restricted to cytoadhesion studies. In addition, parasite invasion and egress or intracellular growth could be investigated. QCM technology is a promising tool for research in basic science on parasite biology and parasite interactions, and can also be applied for research on malaria vaccine and drug development.

## Conclusions

With the presented experiments QCM technology should be made accessible for malaria research. This technology enables the real-time detection of cytoadhesion of iRBCs under flow conditions without affecting viability of parasites. Head-to-head comparison to the standard assays in relevant experimental settings, for example in clinical settings within vaccine trials, are an obvious and important next step that should be taken.

## Additional files

10.1186/s12936-016-1374-7 Example figure of frequency shifts after adding BSA. Incubations with varying concentrations of bovine serum albumin (BSA) (1 mg/ml, 2 %) showed no additional binding events, which would be seen by a continuous drop of frequency. Therefore, no additional unspecific binding sites seem to be accessible.
10.1186/s12936-016-1374-7 Example frequency shift after adding RBCs to a PLL-coated quartz. For this experiment RBCs were added to a PLL-coated quartz. It can clearly be seen that cells attached rapidly (within minutes) to the quartz indicated by a very strong frequency shift (−3389 Hz). This shows that PLL is unspecifically sticky to cells and RBCs attach rapidly to it.
10.1186/s12936-016-1374-7 Example frequency shift of a PLL-coated quartz after adding human serum (HSA) and subsequent addition of iRBCs (FCR3-CSA). For this control experiment, PLL-coated quartzes were incubated with human serum so that proteins bound to PLL. Subsequent addition of iRBCs showed no binding events (no frequency shift) as they do not bind to proteins found in the serum. This controls shows that parasites do not bind unspecifically to any analyte that is coated to PLL.

## Abbreviations

ATCC: American type culture collection
BSA: bovine serum albumin
CSA: chondroitin sulfate A
IFT: indirect immunofluorescence test
iRBC: infected red blood cell
MACS: magnetic cell isolation and cells separation
NEAA: non-essential amino acid solution
PfEMP1: *Plasmodium falciparum* erythrocyte membrane protein 1
PLL: poly-l-lysine
QCM: quartz crystal microbalance
RBC: red blood cell
RT: room temperature
