# Supplementary figures and images for: Real-time measurement of Plasmodium falciparum-infected erythrocyte cytoadhesion with a quartz crystal microbalance
Source: Malar J. 2016 Jun 13;15:317. doi: 10.1186/s12936-016-1374-7 (PMC4906606; doi:10.1186/s12936-016-1374-7)

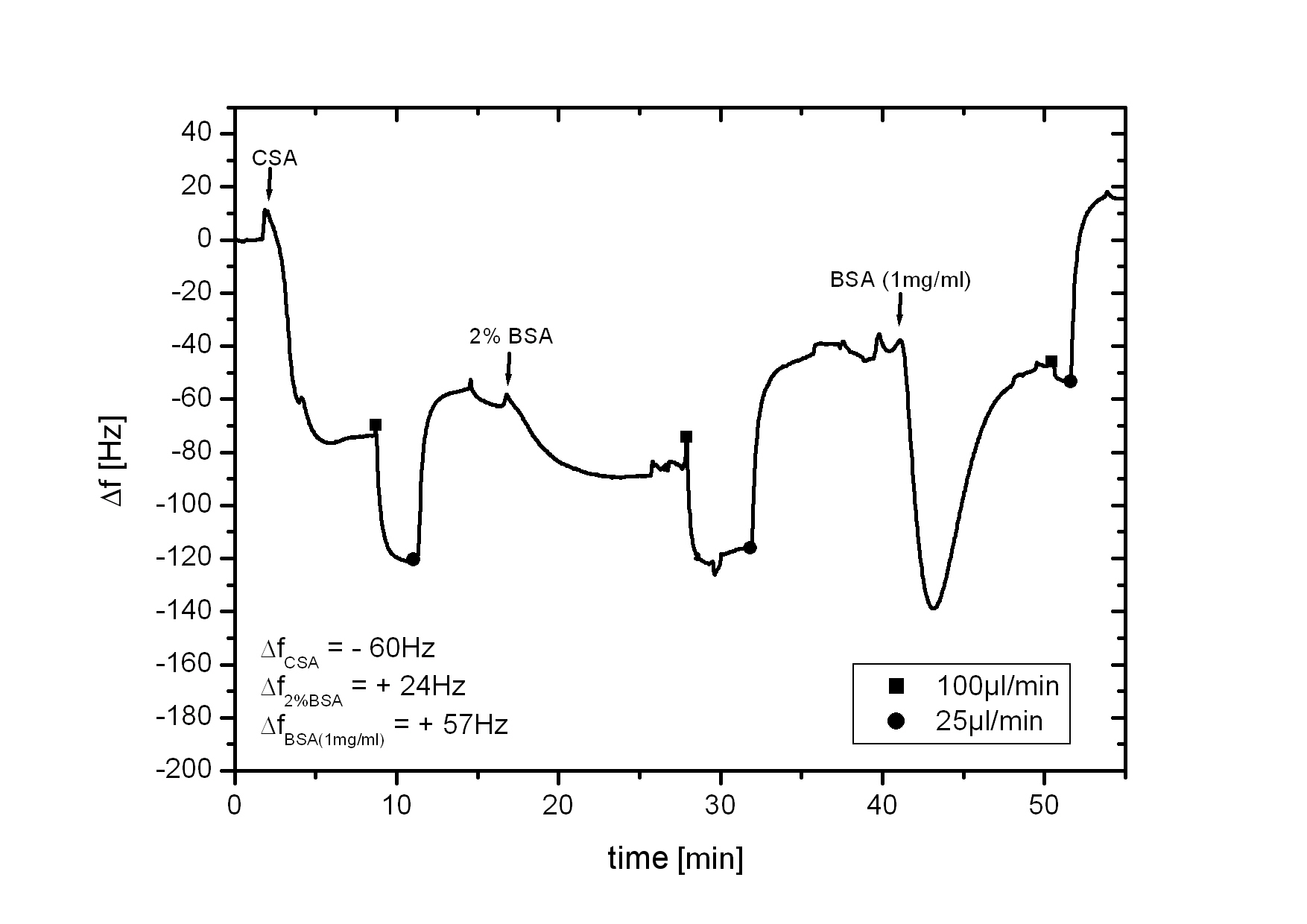

Supplement: Supplementary file 1 — 10.1186/s12936-016-1374-7 Example figure of frequency shifts after adding BSA. Incubations with varying concentrations of bovine serum albumin (BSA) (1 mg/ml, 2 %) showed no additional binding events, which would be seen by a continuous drop of frequency. Therefore, no additional unspecific binding sites seem to be accessible. [file 12936_2016_1374_MOESM1_ESM.tif]

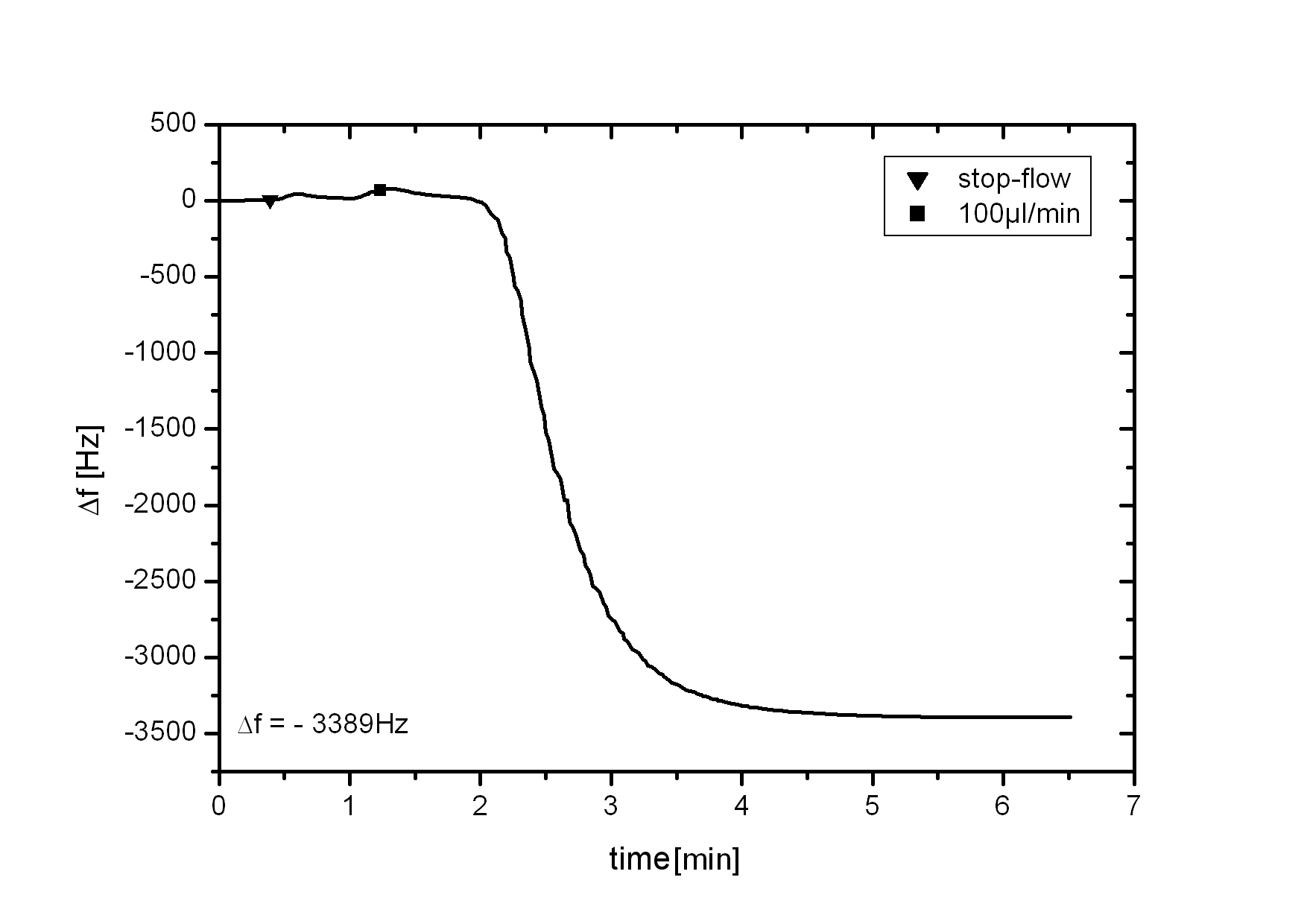

Supplement: Supplementary file 2 — 10.1186/s12936-016-1374-7 Example frequency shift after adding RBCs to a PLL-coated quartz. For this experiment RBCs were added to a PLL-coated quartz. It can clearly be seen that cells attached rapidly (within minutes) to the quartz indicated by a very strong frequency shift (−3389 Hz). This shows that PLL is unspecifically sticky to cells and RBCs attach rapidly to it. [file 12936_2016_1374_MOESM2_ESM.tif]

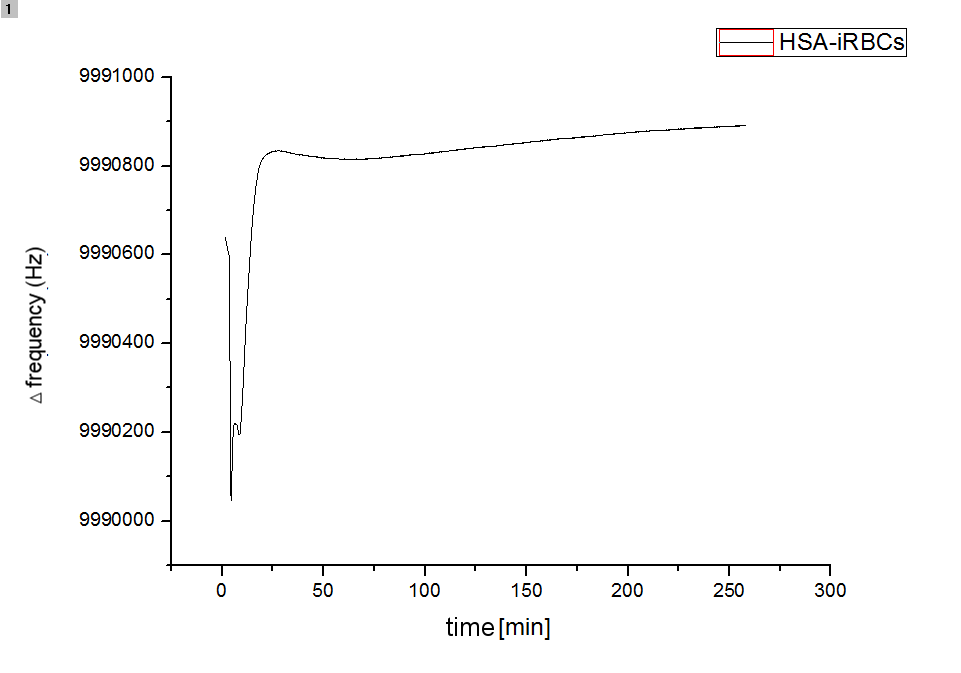

Supplement: Supplementary file 3 — 10.1186/s12936-016-1374-7 Example frequency shift of a PLL-coated quartz after adding human serum (HSA) and subsequent addition of iRBCs (FCR3-CSA). For this control experiment, PLL-coated quartzes were incubated with human serum so that proteins bound to PLL. Subsequent addition of iRBCs showed no binding events (no frequency shift) as they do not bind to proteins found in the serum. This controls shows that parasites do not bind unspecifically to any analyte that is coated to PLL. [file 12936_2016_1374_MOESM3_ESM.tif]
